# Testing the extraction of 12 mycotoxins from aqueous solutions by insoluble beta-cyclodextrin bead polymer

**DOI:** 10.1007/s11356-021-15628-1

**Published:** 2021-07-28

**Authors:** Violetta Mohos, Zelma Faisal, Eszter Fliszár-Nyúl, Lajos Szente, Miklós Poór

**Affiliations:** 1 Department of Pharmacology, Faculty of Pharmacy, University of Pécs, Rókus u. 2, Pécs, H-7624 Hungary; 2 Food Biotechnology Research Group, János Szentágothai Research Centre, University of Pécs, Ifjúság útja 20, Pécs, H-7624 Hungary; 3 CycloLab Cyclodextrin Research & Development Laboratory, Ltd., Illatos út 7, Budapest, H-1097 Hungary

**Keywords:** Mycotoxin, Cyclodextrin, Mycotoxin extraction, Beta-cyclodextrin bead polymer, Mycotoxin binder, Toxin removal

## Abstract

Mycotoxins are toxic metabolites of filamentous fungi; they are common contaminants in numerous foods and beverages. Cyclodextrins are ring-shaped oligosaccharides, which can form host-guest type complexes with certain mycotoxins. Insoluble beta-cyclodextrin bead polymer (BBP) extracted successfully some mycotoxins (e.g., alternariol and zearalenone) from aqueous solutions, including beverages. Therefore, in this study, we aimed to examine the ability of BBP to remove other 12 mycotoxins (including aflatoxin B1, aflatoxin M1, citrinin, dihydrocitrinone, cyclopiazonic acid, deoxynivalenol, ochratoxin A, patulin, sterigmatocystin, zearalanone, α-zearalanol, and β-zearalanol) from different buffers (pH 3.0, 5.0, and 7.0). Our results showed that BBP can effectively extract citrinin, dihydrocitrinone, sterigmatocystin, zearalanone, α-zearalanol, and β-zearalanol at each pH tested. However, for the removal of ochratoxin A, BBP was far the most effective at pH 3.0. Based on these observations, BBP may be a suitable mycotoxin binder to extract certain mycotoxins from aqueous solutions for decontamination and/or for analytical purposes.

## Introduction

**Fig. 1:**
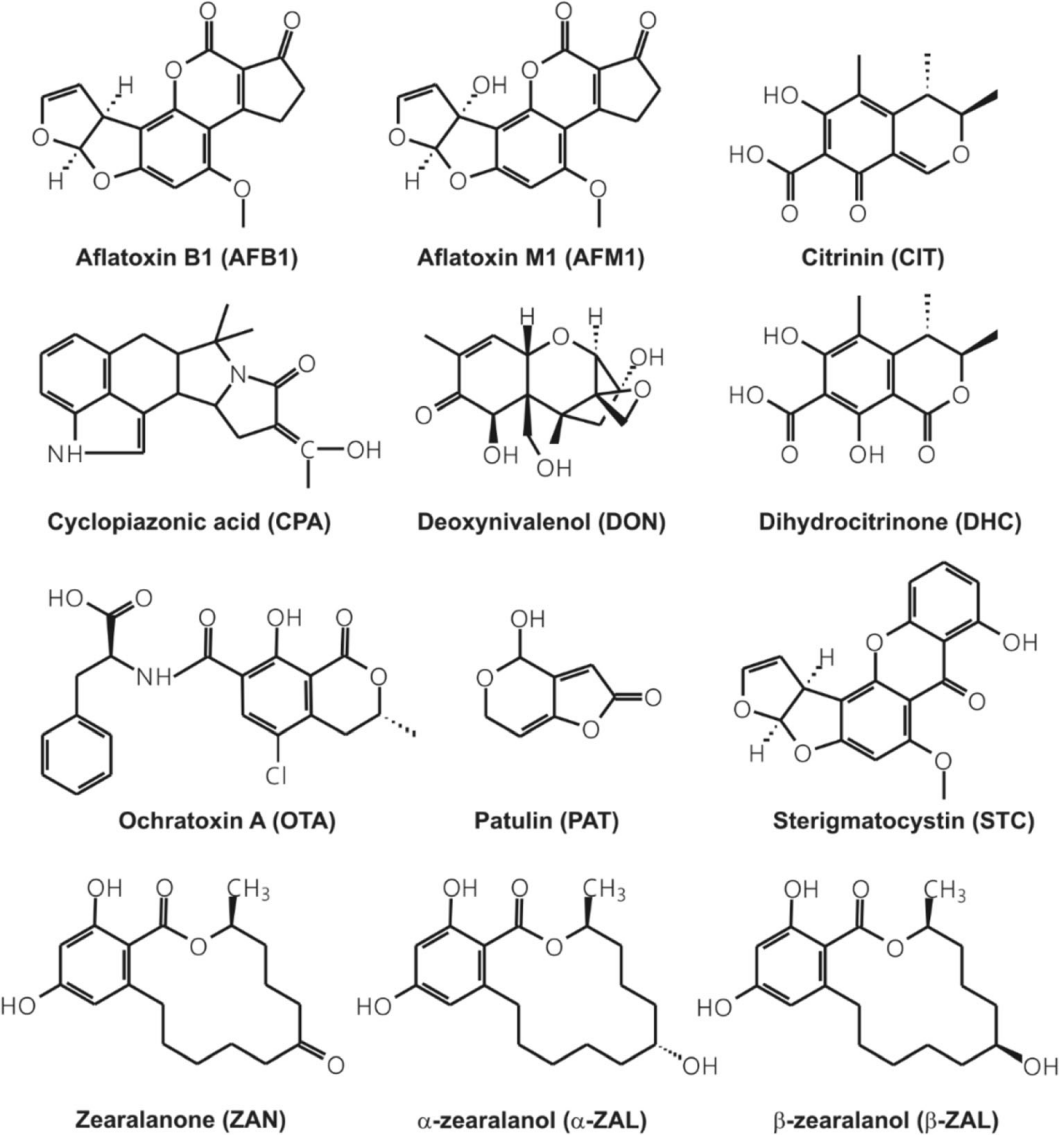
Chemical structures of aflatoxin B1 (AFB1), aflatoxin M1 (AFM1), citrinin (CIT), cyclopiazonic acid (CPA), deoxynivalenol (DON), dihydrocitrinone (DHC), ochratoxin A (OTA), patulin (PAT), sterigmatocystin (STC), zearalanone (ZAN), α-zearalanol (α-ZAL), and β-zearalanol (β-ZAL)

Mycotoxins, the toxic secondary metabolites of filamentous fungi, are common food contaminants (Bennett and Klich 2003; da Rocha et al. 2014). Aflatoxins are mainly produced by *Aspergillus flavus* and *Aspergillus parasiticus*. They were isolated in the 1960s, after the death of more than 100,000 turkeys in “turkey X” disease, due to the consumption of aflatoxin-contaminated peanut meal (Bennett and Klich 2003). Aflatoxins appear in nuts, cereals, figs, vegetables, meat, and spices, possessing primarily hepatotoxic, mutagenic, and carcinogenic effects (Bennett and Klich 2003; da Rocha et al. 2014; Klingelhöfer et al. 2018). The International Agency for Research on Cancer (IARC) classified aflatoxins as Group 1 carcinogens (IARC 2012). Aflatoxin B1 (AFB1) is the most frequent and the most toxic member of this group, while aflatoxin M1 (AFM1) is a metabolite of AFB1 which is a common contaminant in milk (Fig. 1) (Bennett and Klich 2003; Klingelhöfer et al. 2018; Smith and Groopman 2019). Sterigmatocystin (STC; Fig. 1) is a precursor in the biosynthesis of aflatoxins; it exerts mutagenic, carcinogenic, and teratogenic effects and classified as a possible carcinogen (Group 2B) by the IARC (Veršilovskis and De Saeger 2010). STC contaminates typically rapeseed, peanut, spices, and cereals (e.g., wheat, barley, and rice); furthermore, it has also been detected in beer, cocoa, and coffee beans (Veršilovskis and De Saeger 2010). Cyclopiazonic acid (CPA; Fig. 1) was isolated from *Penicillium cyclopium*; nevertheless, several *Penicillium* and *Aspergillus* molds can produce CPA (Bennett and Klich 2003). It appears as a contaminant in oilseeds, cereals, nuts, maize, meat, milk, egg, and peanut (Ostry et al. 2018). The acute toxicity of CPA is low; however, based on animal studies, the chronic exposure to the mycotoxin may cause degenerative changes in the gastrointestinal tract, kidney, liver, and central nervous system (Ostry et al. 2018). Ochratoxin A (OTA) and citrinin (CIT), produced by *Aspergillus*, *Penicillium*, and/or *Monascus* species, are nephrotoxic mycotoxins (Fig. 1) (EFSA 2012; 2020). CIT frequently appears as a contaminant in grains (e.g., wheat, barley, oat, and rye), rice, beans, peas, spices, nuts, and fruits (EFSA 2012), while OTA occurs for example in cereals, fruits, meat, spices, cacao, chocolate, coffee, tea, beer, and wine (EFSA 2020). IARC classified OTA as a possible human carcinogen (Group 2B) (EFSA 2020). Dihydrocitrinone (DHC; Fig. 1) is the major urinary metabolite of CIT, which is less toxic and more hydrophilic than the parent mycotoxin (Ali et al. 2018; Degen et al. 2018). DHC is not a food contaminant; however, we also examined its extraction from buffers, because the cyclodextrin polymer tested may also be suitable for analytical sample preparation regarding body fluids. Patulin (PAT; Fig. 1) is formed by *Aspergillus* and *Penicillium* species. It occurs in different fruits (especially in apple and pear) and in the corresponding products (e.g., fruit juices) (Vidal et al. 2019). Acute PAT intoxication causes gastrointestinal disturbances (e.g., nausea, vomiting, ulceration, and lesions), while its mutagenic, neurotoxic, immunotoxic, genotoxic, teratogenic, and carcinogenic effects have also been reported as a result of the chronic exposure (Puel et al. 2010; Vidal et al. 2019). Deoxynivalenol (DON or vomitoxin; Fig. 1) is a trichothecene mycotoxin produced by *Fusarium* species (e.g., *Fusarium graminearum* and *Fusarium culmorum*) (Ji et al. 2019). DON is one of the most common mycotoxin contaminants in cereals; the exposure can cause gastrointestinal disorders and weight loss as well as the teratogenic and immunotoxic effects of this mycotoxin have also been reported (Ji et al. 2019). Zearalenone is a *Fusarium*-derived mycotoxin; it commonly appears in cereals (e.g., maize, barley, oat, and wheat) and related products (e.g., beer) (EFSA 2017). Despite its non-steroidal structure, zearalenone (and some of its metabolites) binds to estrogen receptors and consequently exerts xenoestrogenic effects; while other harmful (e.g., immunotoxic, nephrotoxic, hepatotoxic, and hematotoxic) impacts are also attributed to this mycotoxin (Ji et al. 2019). The phase I metabolites of zearalenone are α-zearalenol, β-zearalenol, zearalanone (ZAN; Fig. 1), α-zearalanol (α-ZAL; Fig. 1), and β-zearalanol (β-ZAL; Fig. 1) (EFSA 2017). The presence of ZAN and ZALs has been reported in maize products, rice, and soy meal (Ji et al. 2019); while α-ZAL is applied as a growth promoter in certain farm animals in non-EU countries (EFSA 2017). Some zearalenone derivatives, including α-zearalenol and α-ZAL, exert significantly higher estrogenic action than the parent mycotoxin (EFSA 2017).

Cyclodextrins (CD) are ring-shaped oligosaccharides which are extensively utilized by pharmaceutical, food, and cosmetic industries. The most frequently applied CDs are α-, β-, and γ-CDs containing six, seven, and eight glucopyranose units, respectively. CDs have a lipophilic internal cavity which can accommodate non-polar molecules/moieties, while the hydrophilic external part provides them excellent aqueous solubility (Szente and Szemán 2013; Crini 2014). CDs form host-guest type complexes with several mycotoxins, including aflatoxins, alternariol, CIT, OTA, and zearalenone (Dall’Asta et al. 2009; Zhou et al. 2012; Poór et al. 2015a; Wu et al. 2018; Fliszár-Nyúl et al. 2019). Few studies demonstrated that CD technology may be suitable for the extraction/removal of certain mycotoxins (e.g., alternariol, OTA, PAT, zearalenone, and some zearalenone metabolites) from aqueous solutions and/or from beverages (including wine, beer, and apple juice) (Appell and Jackson 2010, 2012; Appell et al. 2018; Poór et al. 2018; Faisal et al. 2019a, 2020; Fliszár-Nyúl et al. 2020).

In the current explorative study, we aimed to investigate the extraction of 12 mycotoxins, namely AFB1, AFM1, CIT, DHC, CPA, DON, OTA, PAT, STC, ZAN, α-ZAL, and β-ZAL (Fig. 1), from different buffers (pH 3.0, 5.0, and 7.0) by insoluble water-swellable β-CD bead polymer (BBP). Our results demonstrate which mycotoxins can be effectively removed from aqueous solution and give a good starting point for the planning of further and deeper investigation regarding the extraction of these mycotoxins from different solutions (including beverages) for decontamination or analytical purposes.

## Materials and methods

### Reagents

All reagents and solvents were analytical or spectroscopic grade. Aflatoxin B1 (AFB1), citrinin (CIT), cyclopiazonic acid (CPA), deoxynivalenol (DON), ochratoxin A (OTA), patulin (PAT), sterigmatocystin (STC), zearalanone (ZAN), α-zearalanol (α-ZAL), and β-zearalanol (β-ZAL) were purchased from Sigma-Aldrich (Waltham, MA, USA). Aflatoxin M1 (AFM1) and dihydrocitrinone (DHC) were obtained from Apollo Scientific (Cheshire, UK) and AnalytiCon Discovery (Potsdam, Germany), respectively. Insoluble water-swellable β-CD bead polymer (BBP; β-cyclodextrin-epichlorohydrin cross-linked bead polymer; β-CD content: 50 m/m%) (Poór et al. 2018; Faisal et al. 2019a; Fliszár-Nyúl et al. 2019) was provided by CycloLab Cyclodextrin Research & Development Laboratory, Ltd. (Budapest, Hungary). Stock solutions of CIT, DHC, OTA, ZAN, and ZALs were prepared in ethanol (96 v/v%, spectroscopic grade; VWR, Debrecen, Hungary), while AFB1, AFM1, CPA, DON, PAT, and STC were dissolved in dimethyl sulfoxide (DMSO, spectroscopic grade; Fluka, Bucharest, Romania). Mycotoxin stock solutions (each 5 mM) were stored at −20 °C.

### Mycotoxin extraction

To test the removal of mycotoxins by BBP, mycotoxin solutions (2 μM, 1.5 mL) were added to increasing amounts (0.0, 1.0, 2.5, 5.0, 10.0, and 20.0 mg) of BBP (final concentrations: 0.0, 0.67, 1.67, 3.33, 6.67, and 13.3 mg/mL) in sodium acetate (0.05 M, pH 5.0) buffer. Samples were incubated in a thermomixer (40 min, 1000 rpm, 25 °C), after which BBP was sedimented by pulse centrifugation (6 s, 4000 g, room temperature). Then, a 500 μL aliquot of supernatants was removed and analyzed by high-performance liquid chromatography (HPLC).

To examine the impact of the environmental pH on mycotoxin removal, the same experiments were performed at pH 3.0 (0.05 M sodium phosphate) and pH 7.0 (0.05 M sodium phosphate), applying 0.0, 1.67, and 6.67 mg/mL final BBP concentrations. However, these buffers interfered with the efficiency of the HPLC method applied for the analyses of ZAN and ZALs. Therefore, the latter mycotoxins were incubated in sodium tartrate (0.05 M, pH 3.0) and TRIS-HCl (0.05 M, pH 7.0) buffers.

Most of the supernatants were directly injected into the HPLC after the sedimentation of BBP. Nevertheless, pH adjustment of certain samples was reasonable for the appropriate conditions of HPLC analyses. The 500 μL aliquots of these supernatants were acidified or alkalinized based on the followings. The pH 3.0 AFM1 supernatants were alkalinized with 3 μL of 1 M NaOH. The pH 5.0 and pH 7.0 CIT samples were acidified with 8 and 10 μL of 1.5 M HCl, respectively. Similarly, pH 7.0 DHC supernatants were acidified with 10 μL of 1.5 M HCl. The pH 3.0 CPA samples were alkalinized with 5 μL of 0.5 M NaOH. The pH 7.0 DON supernatants were acidified with 8 μL of 1.5 M HCl. OTA samples were alkalinized with 7 μL of 3 M NaOH (pH 3.0 samples) or 7 μL of 1 M NaOH (pH 5.0 and pH 7.0 samples). The pH 3.0 STC supernatants were alkalinized with 3 μL of 1 M NaOH, while pH 7.0 STC samples were acidified with 8 μL of 1.5 HCl.

At pH 3.0 (0.05 M sodium phosphate buffer), the interaction of OTA with BBP was quantitatively evaluated employing the Langmuir and Freundlich isotherms (Appell and Jackson 2012; Faisal et al. 2019a; Fliszár-Nyúl et al. 2019). Increasing concentrations of OTA (0.1, 0.2, 0.5, 1.0, 2.5, 5.0, 7.5, and 10 μM) were added to standard amount of BBP (2.0 mg/mL), after which the incubation and sample preparation were performed as described above.

### HPLC analyses

CIT, DHC, and OTA were analyzed by an integrated HPLC system (Jasco, Tokyo, Japan), which included an autosampler (AS-4050), a binary pump (PU-4180), and a fluorescence detector (FP-920). Chromatograms were evaluated using ChromNAV software (Jasco, Tokyo, Japan). Furthermore, AFB1, AFM1, CPA, DON, PAT, STC, ZAN, and ZALs were analyzed by an integrated HPLC system built up from a Waters 510 HPLC pump (Milford, MA, USA), a Rheodyne 7125 injector (Berkeley, CA, USA) with a 20-μL sample loop, and a Waters 486 UV detector (Milford, MA, USA). Chromatograms were evaluated employing Millennium Chromatography Manager software (Waters, Milford, MA, USA). Each HPLC analysis was performed with isocratic elution using 1.0 mL/min flow rate at room temperature, and 20 μL volume of samples was injected.

CIT and DHC samples were driven through a Phenomenex (C18, 4 × 3 mm; Torrance, CA, USA) guard column linked to a Mediterranea SEA18 (C18, 250 × 4.6 mm, 5 μm; Teknokroma, Barcelona, Spain) analytical column. The mobile phase consisted of acetonitrile (HPLC grade; VWR, Debrecen, Hungary), phosphoric acid (pH 3.0), and isopropanol (HPLC grade; VWR, Debrecen, Hungary) (45:45:10 v/v%). CIT and DHC were detected at 505 (*λ*_ex_ = 330 nm) and 420 nm (*λ*_ex_ = 325 nm), respectively.

OTA samples were driven through a Phenomenex (C18, 4 × 3 mm; Torrance, CA, USA) guard column connected to a Kinetex-EVO (C18, 150 × 4.6 mm, 5 μm; Phenomenex, Torrance, CA, USA) analytical column. The eluent contained 0.01 M sodium borate buffer (pH 10.0) and acetonitrile (87:13 v/v%), and OTA was detected at 446 nm (*λ*_ex_ = 383 nm).

AFB1 and AFM1 samples were driven through a Nova-Pak (C18, 20 × 3.9 mm, 4 μm; Waters, Milford, MA, USA) guard column coupled to a Nova-Pak (C18, 150 × 3.9 mm, 4 μm; Waters, Milford, MA, USA) analytical column. Water, methanol (HPLC grade; VWR, Debrecen, Hungary), and acetonitrile (55:30:15 v/v%) were applied as mobile phase. AFB1 and AFM1 were detected at 362 nm.

CPA samples were driven through a Phenomenex (C8, 4 × 3 mm; Torrance, CA, USA) guard column linked to a Mediterranea SEA8 (C8, 150 × 4.6 mm, 5 μm; Teknokroma, Barcelona, Spain) analytical column. The elution was carried out with 0.01 M sodium phosphate buffer (pH 7.0) and acetonitrile (73:27 v/v%). CPA was detected at 280 nm.

DON samples were driven through a Phenomenex (C18, 4 × 3 mm; Torrance, CA, USA) guard column coupled to a Gemini-NX (C18, 150 × 4.6 mm, 3 μm; Phenomenex, Torrance, CA, USA) analytical column. The separation was performed applying water and acetonitrile (80:20 v/v%) as mobile phase. DON was detected at 225 nm.

PAT samples were driven through a Phenomenex (C18, 4 × 3 mm; Torrance, CA, USA) guard column linked to a Kinetex-XB (C18, 250 × 4.6 mm, 5 μm; Phenomenex, Torrance, CA, USA) analytical column. The mobile phase contained water and acetonitrile (90:10 v/v%). PAT was detected at 276 nm.

STC samples were driven through a Phenomenex (C18, 4 × 3 mm; Torrance, CA, USA) guard column linked to a Gemini-NX (C18, 150 × 4.6 mm, 3 μm; Phenomenex, Torrance, CA, USA) analytical column. The mobile phase contained acetonitrile and 0.01 M sodium phosphate buffer (pH 4.55) (50:50 v/v%). STC was detected at 331 nm.

ZAN and ZAL samples were driven through a Phenomenex (C18, 4 × 3 mm; Torrance, CA, USA) guard column coupled to a Mediterranea SEA18 (C18, 250 × 4.6 mm, 5 μm; Teknokroma, Barcelona, Spain) analytical column. The elution was carried out with acetonitrile and water (60:40 v/v%). ZAN and ZALs were detected at 262 nm.

### Statistics

Data represent mean ± SEM values at least from three independent measurements. One-way ANOVA with Tukey’s post hoc test was applied to establish the statistical significance (*p* < 0.01), employing SPSS Statistics software (version 24; IBM, Armonk, NY, USA).

## Results

### Extraction of mycotoxins from sodium acetate buffer (pH 5.0) by BBP

To test the mycotoxin binding of BBP, increasing amounts of the polymer were added to standard concentration of mycotoxins (each 2 μM in 1.5 mL volume) in sodium acetate buffer (pH 5.0).

**Fig. 2:**
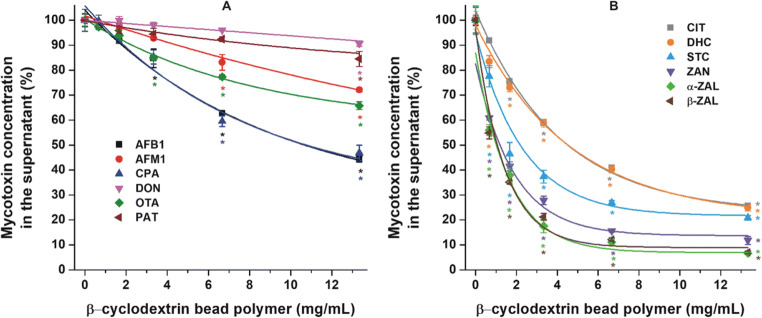
Extraction of mycotoxins from sodium acetate buffer (0.05 M, pH 5.0) by BBP (*p < 0.01). **a**: Mycotoxins which were extracted with less than 75% efficacy by 13.3 mg/mL (or 20.0 mg/1.5 mL) BBP; **b**: mycotoxins which were extracted with 75% or even better efficacy by 13.3 mg/mL BBP

Fig. 2a demonstrates the mycotoxins which were extracted with less than 75% efficacy by 13.3 mg/mL (or 20.0 mg/1.5 mL) BBP. The bead polymer barely affected DON and PAT contents of the solutions. Furthermore, approximately 28 and 35% decreases in the concentrations of AFM1 and OTA were caused by 13.3 mg/mL BBP, respectively. In addition, more than 50% of AFB1 and CPA were removed by the same amount of the polymer.

Fig. 2b represents the mycotoxins which were extracted with 75% or even better efficacy by 13.3 mg/mL BBP. Among these mycotoxins, approximately 75% of CIT and DHC were extracted, followed by STC (80%). Interestingly, the lower concentrations of BBP (0.67 to 3.33 mg/mL) induced a much steeper decrease in the STC content of the solution compared to CIT and DHC (Fig. 2b). Moreover, BBP proved to be the strongest binder of ZAN and ZALs, removing approximately 90–95% of these mycotoxins at 13.3 mg/mL concentration.

### Testing the pH dependence of mycotoxin extraction

**Fig. 3:**
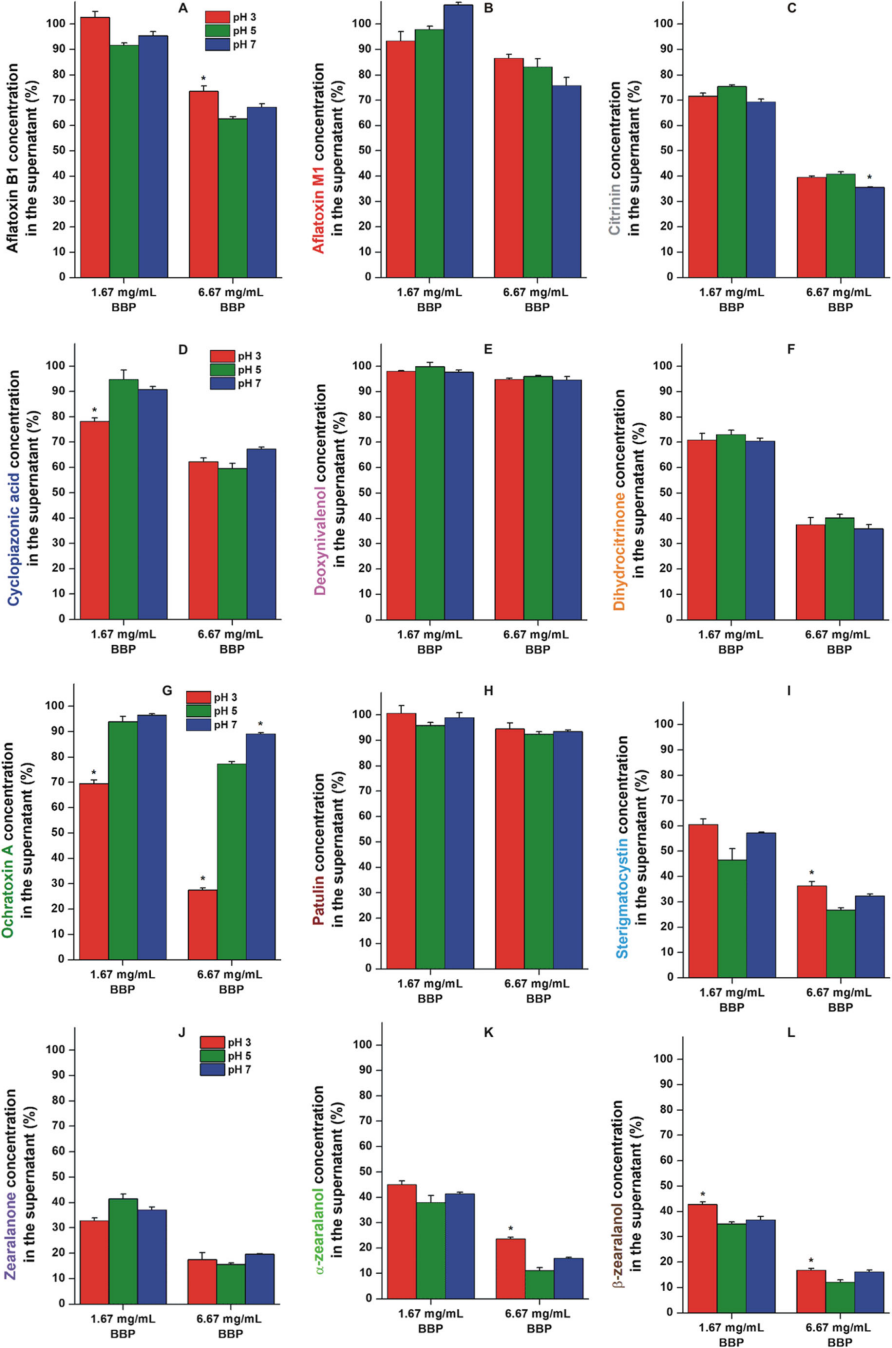
Extraction of **a** AFB1; **b** AFM1; **c** CIT; **d** CPA; **e** DON; **f** DHC; **g** OTA; **h** PAT; **i** STC; **j** ZAN; **k** α-ZAL; **l** β-ZAL from different buffers (pH 3.0, pH 5.0, and pH 7.0; see further details in “Mycotoxin Extraction” section) by 1.67 and 6.67 mg/mL BBP (*p < 0.01: statistical significance of pH 3.0 and pH 7.0 vs. pH 5.0 samples)

**Fig. 4:**
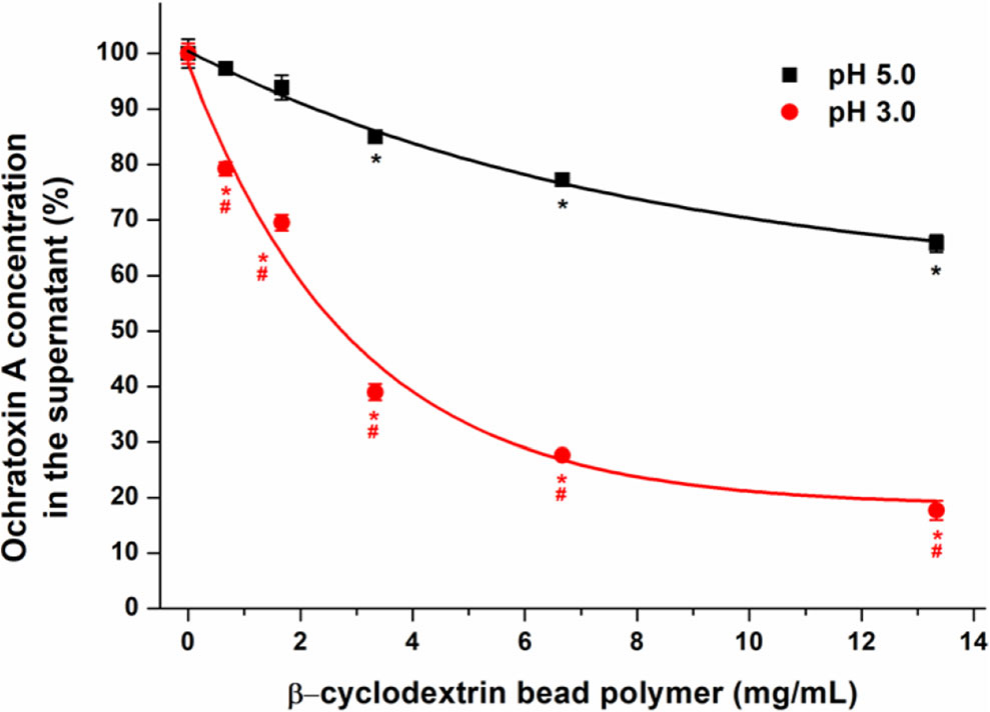
Extraction of OTA (2 μM) from 0.05 M sodium phosphate (pH 3.0) and 0.05 M sodium acetate (pH 5.0) buffers by BBP (*p < 0.01: compared to the control; #*p* < 0.01: compared to the data measured at pH 5.0)

The pH dependence regarding the mycotoxin binding of BBP was also examined. Since the pH of beverages is typically in the acidic or neutral range (Feldman and Barnett 1995), we tested the mycotoxin extraction between pH 3.0 and pH 7.0 (see details in “Mycotoxin Extraction” section). Fig. 3 illustrates the removal of mycotoxins at pH 3.0, pH 5.0, and pH 7.0 by 1.67 and 6.67 mg/mL BBP. Under the applied conditions, we did not find significant differences regarding AFM1, DON, DHC, PAT, and ZAN (Fig. 3). CIT was the only mycotoxin where a little bit higher mycotoxin removal was observed at pH 7.0 vs. pH 5.0; however, only the higher BBP concentration caused statistically significant difference (Fig. 3c). Furthermore, the slightly lower removal of AFB1 (6.67 mg/mL BBP), STC (6.67 mg/mL BBP), α-ZAL (6.67 mg/mL BBP), and β-ZAL (1.67 and 6.67 mg/mL BBP) was noticed at pH 3.0 than at pH 5.0. In the presence of 1.67 mg/mL BBP, the decrease in CPA content was the largest at pH 3.0; however, we did not observe pH-dependent differences when 6.67 mg/mL polymer concentration was applied. Despite the above-listed statistically significant differences regarding the mycotoxin removal in different buffers, the only relevant pH effect was demonstrated by OTA. The decrease in the pH to 3.0 considerably enhanced the removal of OTA by BBP compared to both pH 5.0 and pH 7.0 (Fig. 3g). Therefore, the extraction of OTA at pH 3.0 was also tested with each﻿ BBP concentration applied in “Mycotoxin Extraction” section. As it is demonstrated in Fig. 4, the lower pH favors the interaction of OTA with BBP, leading to the strong decrease in the mycotoxin content at pH 3.0 and resulting in more than 80% removal of OTA by 13.3 mg/mL BBP.

### Evaluation of OTA-BBP interaction at pH 3.0 employing the Langmuir and Freundlich isotherms

**Fig. 5:**
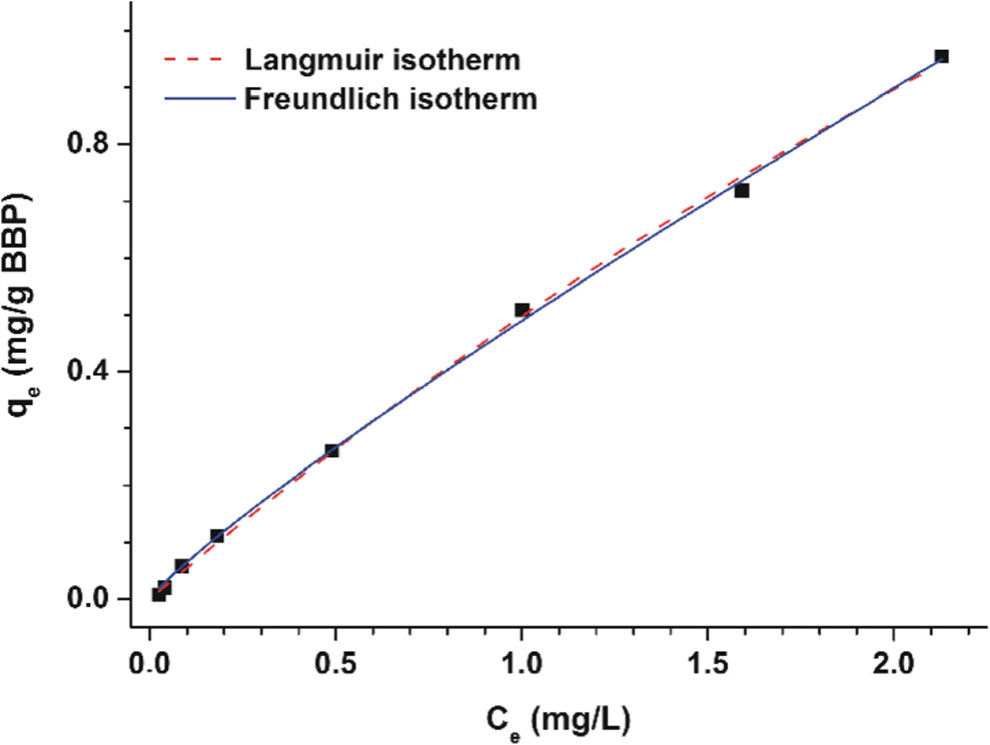
Langmuir (dashed red line) and Freundlich (solid blue line) isotherms of OTA-BBP interaction in sodium phosphate buffer (0.05 M, pH 3.0), where *q*_e_ is the amount of bound OTA (mg) by BBP (g) , while *C*_e_ means the amount of unbound OTA (mg/L) in the solution at equilibrium (see further details in “Mycotoxin Extraction ” section)

Both Langmuir (*R*^2^ = 0.999) and Freundlich (*R*^2^ = 0.999) models showed excellent fitting with the experimental data (Fig. 5). The Langmuir equilibrium constant (*K*_L_) was 0.12 ± 0.03 L/mg, and the maximum quantity of OTA (mg) bound per gram of BBP (*Q*_0_) was 4.50 ± 0.89 mg/g. The Freundlich constant (*K*_F_) and the 1/*n* value (*n* is the heterogeneity index) were 0.49 ± 0.01 (mg/g) × (L/mg)^1/*n*^ and 0.88 ± 0.02, respectively.

## Discussion

Few studies demonstrated that BBP may be a promising candidate for the removal of some mycotoxins from aqueous solutions: For example, alternariol and zearalenone have been successfully extracted from buffers and from certain beverages (wine and beer, respectively) (Poór et al. 2018; Fliszár-Nyúl et al. 2019, 2020). Therefore, in the current explorative study, we aimed to examine the ability of BBP to extract other 12 mycotoxins (AFB1, AFM1, CIT, CPA, DON, DHC, OTA, PAT, STC, ZAN, α-ZAL, and β-ZAL) from aqueous buffers. Mycotoxins are common contaminants in food and beverages (e.g., milk, coffee, beer, wine, and fruit juices) (Bennett and Klich 2003; Veršilovskis and De Saeger 2010). Since the pH of these drinks are typically acidic or neutral (Feldman and Barnett 1995), our experiments were performed between pH 3.0 and pH 7.0.

The host-guest type complex formation of several mycotoxins with CD “monomers” has been widely studied. Aflatoxins (Dall’Asta et al. 2003; Aghamohammadi and Alizadeh 2007; Wu et al. 2018), CIT (Zhou et al. 2012; Poór et al. 2016), DHC (Faisal et al. 2019b), and OTA (Hashemi and Alizadeh 2009; Poór et al. 2015a) form complexes with native and chemically modified β- and/or γ-CDs; however, these mycotoxins bind to the native β-CD with relatively low affinity (*K* = 10^2^ to 10^3^ L/mol). However, dianionic OTA forms highly stable (*K* > 10^4^ L/mol) complexes with (2-hydroxy-3-*N,N,N*-trimethylamino)propyl-beta-CD (Poór et al. 2015a). Under acidic and neutral conditions, zearalenone also binds to β-CDs with high affinity (*K* = 10^4^ to 10^5^ L/mol) (Dall’Asta et al. 2008, 2009; Poór et al. 2015b). To the best of our knowledge, the complexation of CPA, DON, PAT, STC, ZAN, α-ZAL, and β ZALs has not been investigated with any CDs.

Only limited data are available regarding the interaction of mycotoxins with CD polymers. The successful extraction of OTA and PAT by polyurethane-β-CD polymer has been reported from aqueous solutions, including wine and apple juice, respectively (Appell and Jackson 2010, 2012). In addition, BBP considerably decreased the mycotoxin (alternariol, zearalenone, α-zearalenol, β-zearalenol, zearalenone-14-glucoside, and zearalenone-14-sulfate) content of aqueous solutions and effectively removed alternariol and zearalenone from wine and from beer samples, respectively (Poór et al. 2018; Faisal et al. 2019a, 2020; Fliszár-Nyúl et al. 2019, 2020). At pH 5.0, BBP produced the highest removal of ZAN and ZALs; however, it seems to be a suitable binder of CIT, DHC, and STC as well (Fig. 2b). No data are available regarding the interactions of ZAN, ZALs, and STC with CDs; however, zearalenone forms stable complexes with β-CD (*K* ≈ 10^4^ L/mol) and was successfully removed from aqueous solutions by BBP (Poór et al. 2018). Nevertheless, the binding constants of CIT-β-CD and DHC-β-CD complexes are low (*K* ≈ 10^2^ L/mol) (Zhou et al. 2012; Poór et al. 2016; Faisal et al. 2019b); therefore, the removal of these mycotoxins by BBP is unexpectedly high. Similar phenomenon was observed at pH 3.0 with OTA (Fig. 4), despite the fact that the mycotoxin forms poorly stable complexes with β-CD (*K* ≈ 10^2^ L/mol) (Poór et al. 2015a). In addition, Verrone et al. reported the highest affinity of β-CD towards dianionic OTA (both carboxyl and phenolic hydroxyl groups are deprotonated), followed by the nonionized and the monoanionic (only the carboxyl group is deprotonated) forms (Verrone et al. 2007). These results indicate the cooperative interactions of CD rings in BBP with CIT, DHC, and OTA, as it has also been observed regarding mycotoxin alternariol and some other compounds (Harada et al. 1976; Saenger 1980; Fliszár-Nyúl et al. 2019). Under the applied conditions, BBP (13.3 mg/mL) removed approximately 50% of AFB1 and CPA; however, the polymer only slightly decreased the concentrations of AFM1, PAT, and DON (Fig. 2a).

The pH dependence of mycotoxin extraction was tested in the pH range 3.0 to 7.0. No or only slight changes were observed in the extraction of mycotoxins tested, except OTA (Fig. 3). BBP strongly decreased the OTA content of the solution at pH 3.0 (Figs. 3g and 4.). These data suggest that BBP mainly interacts with the nonionic form of OTA, which is also supported by the effective removal of the mycotoxin from red wine by polyurethane-β-CD polymer (Appell and Jackson 2012). Amadasi et al. suggest the inclusion of the phenyl ring of L-phenylalanine in the CD cavity (Amadasi et al. 2007). Furthermore, the protruding parts of OTA (the carboxyl group and the isocoumarin moiety) form hydrogen bonds with the outer hydroxyl groups of the CD, which can further stabilize the inclusion (Amadasi et al. 2007). The carboxyl and phenolic hydroxyl groups of OTA can be ionized (acid dissociation constants are 4.2–4.4 and 7.0–7.3, respectively) (Perry et al. 2003). The deprotonation of the carboxyl and/or the phenolic hydroxyl group(s) at higher pH (e.g., pH 5.0 and pH 7.0) may explain the lower efficacy of BBP regarding OTA removal.

The Langmuir and Freundlich sorption isotherms are suitable for the quantitative evaluation of mycotoxin-BBP interactions (Appell and Jackson 2012; Faisal et al. 2019a; Fliszár-Nyúl et al. 2019). The Langmuir model typically characterizes a strictly homogenous monolayer adsorption, while the Freundlich isotherm does not need this restriction (Ayawei et al. 2017). Based on the Freundlich model, the heterogeneity index (*n*) was close to one, indicating the relatively homogenous sorption of OTA by BBP. In our previous studies, the extraction of zearalenone (Poór et al. 2018) and alternariol (Fliszár-Nyúl et al. 2019) was also tested from aqueous buffers by BBP. Regarding this polymer, the *Q*_0_ values of OTA and zearalenone were similar, while it was significantly higher for alternariol. However, both the Langmuir equilibrium constant (*K*_L_) and the adsorptive capacity (*K*_F_, determined applying the Freundlich model) demonstrate the weaker interaction of BBP with OTA (*K*_L_ = 0.12 L/mg; *K*_F_ = 0.49 (mg/g) × (L/mg)^1/*n*^) compared to zearalenone (*K*_L_ = 0.60 L/mg; *K*_F_ = 1.16 (mg/g) × (L/mg)^1/*n*^) and alternariol (*K*_L_ = 0.16 L/mg; *K*_F_ = 5.52 (mg/g) × (L/mg)^1/*n*^). These data are also in agreement with our observations that the removal of OTA by BBP is less effective vs. zearalenone or alternariol (Poór et al. 2018; Fliszár-Nyúl et al. 2019).

For comparison of the mycotoxin binding ability of BBP with other CD polymers, adsorbents, and nanoparticles, our results were combined with previously reported data in Table 1, including the mycotoxin binder used, the mycotoxin extracted, the environmental conditions, and the toxin removal.

**Table 1:** Mycotoxin removal by CD polymers, adsorbents, and nanoparticles: comparison of BBP with other mycotoxin binders

**Mycotoxin binder**	**Mycotoxin**	**Matrix**	**Concentration of the mycotoxin binder used**	**Toxin removal**	**References**
Silicon carbide nanoparticles	AFB1	Aqueous solution (1 g/L, pH 9.0)	40 mg (volume was not indicated)	46 μg AFB1/mg adsorbent	Gupta et al. 2017
Iron nanoparticles	AFB1	Aqueous solution (pH 9.0)	1.0 mg/mL	131–139 ng AFB1/mg adsorbent (85–90% at pH 9.0)	Asghar et al. 2018
***BBP***	***AFB1***	***Acetate buffer*** ***(pH 5.0)***	***13.3 mg/mL***	***56%***	***Current study***
Bentonite (with trioctahedral smectite)	AFM1	Milk	25 mg/mL	~100% (reduced below limit of detection)	Carraro et al. 2014
Aptamer magnetic nanoparticles	AFM1	Milk	1.1 mg/mL	98–112%	Khodadadi et al. 2018
***BBP***	***AFM1***	***Acetate buffer*** ***(pH 5.0)***	***13.3 mg/mL***	***28%***	***Current study***
1,4-dihydroxy-2-naphthoic acid molecularly imprinted polymer	CIT	Corn extract	300 mg (solid phase extraction cartridge)	82–92%	Appell et al. 2015
Organo-modified bentonite	CIT	Water	1.0 mg/mL	1.84 μg CIT/mg polymer	Saeed et al. 2020
***BBP***	***CIT***	***Acetate buffer*** ***(pH 5.0)***	***13.3 mg/mL***	***74%***	***Current study***
Acidic clay	CPA	Water	10 mg/mL	95%	Dwyer et al. 1997
***BBP***	***CPA***	***Acetate buffer*** ***(pH 5.0)***	***13.3 mg/mL***	***53%***	***Current study***
Yeast cell wall	DON	Phosphate buffer (pH 6.0)	5.0 mg/mL	23%	Kong et al. 2014
Pillared montmorillonite	DON	Aqueous solutions (pH 2.0 and 6.8)	0.5 mg/mL	29% (pH 2.0) 34% (pH 6.8)	Zhang et al. 2021
Magnetic nanostructured particles	DON	Water wort	211 mg/mL	26% (water) ~20% (wort)	González-Jartín et al. 2019
***BBP***	***DON***	***Acetate buffer*** ***(pH 5.0)***	***13.3 mg/mL***	***9%***	***Current study***
***BBP***	***DHC***	***Acetate buffer*** ***(pH 5.0)***	***13.3 mg/mL***	***75%***	***Current study***
Activated carbon	OTA	PBS wine	1.0 mg/mL	100%	Var et al. 2008
β-CD-polyurethane polymer	OTA	Phosphate buffers pH 3.5 pH 7.0 pH 9.5 Wine	2.0 mg/mL	95–100% (pH 3.5) 95–100% (pH 7.0) 57–80% (pH 9.5) 88–95% (wine)	Appell and Jackson 2012
Polyvinyl-polypyrrolidone	OTA	Red wine	0.5 mg/mL	40%	Quintela et al. 2012
Chitosan	OTA	Red wine	5.0 mg/mL	67%	Quintela et al. 2012
Calcium alginate beads	OTA	Grape juice	1 mL suspension/25 mL juice	>80%	Farbo et al. 2016
***BBP***	***OTA***	***Phosphate buffer (pH 3.0)***	***13.3 mg/mL***	***82%***	***Current study***
Tolylene 2,4-diisocyanate crosslinked β-CD polymer	PAT	Acetate buffer (pH 5.5) ethanol acetonitrile	10 mg/mL	29 μg PAT/ mg polymer 7.3 μg PAT/ mg polymer 6.7 μg PAT/ mg polymer	Appell and Jackson 2010
Magnetic chitosan	PAT	Kiwi juice	10 mg/mL	19.4 μg PAT/ mg polymer (96%)	Luo et al. 2016
***BBP***	***PAT***	***Acetate buffer*** ***(pH 5.0)***	***13.3 mg/mL***	***15%***	***Current study***
Egyptian montmorillonite	STC	Aqueous solutions (pH 2.0 and 10.0), distilled water	0.5–4.0 mg/L	93–98%	Abdel-Wahhab et al. 2005
***BBP***	***STC***	***Acetate buffer*** ***(pH 5.0)***	***13.3 mg/mL***	***79%***	***Current study***
Core-shell poly(dopamine) magnetic nanoparticles	ZAN	Milk	3.2 mg/mL	99%	González-Sálamo et al. 2017
***BBP***	***ZAN***	***Acetate buffer*** ***(pH 5.0)***	***13.3 mg/mL***	***88%***	***Current study***
Core-shell poly(dopamine) magnetic nanoparticles	α-ZAL	Milk	3.2 mg/mL	100%	González-Sálamo et al. 2017
***BBP***	***α-ZAL***	***Acetate buffer*** ***(pH 5.0)***	***13.3 mg/mL***	***93%***	***Current study***
Core-shell poly(dopamine) magnetic nanoparticles	β-ZAL	Milk	3.2 mg/mL	82%	González-Sálamo et al. 2017
***BBP***	***β-ZAL***	***Acetate buffer*** ***(pH 5.0)***	***13.3 mg/mL***	***93%***	***Current study***

In conclusion, the extraction of 12 mycotoxins by BBP was tested in different buffers (pH 3.0, 5.0, and 7.0). BBP induced the concentration-dependent decrease in the mycotoxin content and proved to be an effective binder of CIT, DHC, OTA, STC, ZAN, and ZALs. Among the mycotoxins tested, only the extraction of OTA showed considerable pH dependence: its removal by BBP was far the most effective at pH 3.0. Our results suggest that BBP may be a suitable mycotoxin binder to extract certain mycotoxins from aqueous solutions for decontamination and/or for analytical purposes.
